# Dual‐Enhanced Doping in ReSe_2_ for Efficiently Photoenhanced Hydrogen Evolution Reaction

**DOI:** 10.1002/advs.202000216

**Published:** 2020-03-16

**Authors:** Ran Wang, Jiecai Han, Ping Xu, Tangling Gao, Jun Zhong, Xianjie Wang, Xinghong Zhang, Zhijun Li, Lingling Xu, Bo Song

**Affiliations:** ^1^ National Key Laboratory of Science and Technology on Advanced Composites in Special Environments Harbin Institute of Technology Harbin 150001 China; ^2^ School of Chemistry and Chemical Engineering Harbin Institute of Technology Harbin 150001 China; ^3^ Institute of Petrochemistry Heilongjiang Academy of Sciences Harbin 150040 China; ^4^ Institute of Functional Nano and Soft Materials Soochow University Suzhou Jiangsu 215123 China; ^5^ Department of Physics Harbin Institute of Technology Harbin 150001 China; ^6^ Shanghai Synchrotron Radiation Facility Shanghai Institute of Applied Physics Shanghai 201800 China; ^7^ Key Laboratory for Photonic and Electronic Bandgap Materials Ministry of Education School of Physics and Electronic Engineering Harbin Normal University Harbin 150025 China

**Keywords:** dual‐enhanced doping, photoenhanced hydrogen evolution reaction (PE‐HER), ReSe _2_ nanosheets

## Abstract

Rhenium dichalcogenides (ReX_2_, X = S or Se) are catalysts that have great promise for the photoenhanced hydrogen evolution reaction (PE‐HER) because of their unique physiochemical properties. However, the catalytic performance is still restricted by their low concentration of electrocatalytic activity sites and poor injection of hot electrons. Herein, dual‐enhancement in ReSe_2_ nanosheets (NSs) with high concentration of active sites and efficient use of hot electrons is simultaneously achieved with moderate Mo doping. Contributions from exposed catalytically active sites, improved electrical conductivity, and enhanced solar spectral response are systematically investigated. Superior PE‐HER catalytical performance is obtained in Re_0.94_Mo_0.06_Se_2_, which has more catalytically active sites and optimized band structure than other Re_1−_ *_x_*Mo*_x_*Se_2_ samples. Here, it is demonstrated that only doping can reduce the overpotential (η_10_) from 239 to 174 mV at −10 mA cm^−2^ (Δ1η_10_ = 65 mV). Then, η_10_ is further improved to 137 mV under simulated AM 1.5 sun illumination (Δ2η_10_ = 37 mV). The total improvement (Δη_10_) toward PE‐HER is 102 mV (Δ1η_10_ + Δ2η_10_ = 102 mV) in optimal Re_0.94_Mo_0.06_Se_2_. This work presents a new perspective for researching high‐efficiency photoenhanced HER ReSe_2_‐based electrocatalysts and other layered transition metal dichalcogenides.

## Graphical abstract

Dual enhancement with high concentration of active sites and efficient use of hot electrons is simultaneously achieved in moderate Mo doping ReSe_2_ nanosheets for the photoenhanced hydrogen evolution reaction (HER). The optimal Re_0.94_Mo_0.06_Se_2_ exhibits a high‐efficiency HER catalytic performance with the total improvement (Δη_10_) of 102 mV at −10 mA cm^−2^.

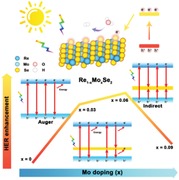

Preparation of high‐efficiency electrocatalysts for the hydrogen evolution reaction (HER) is pivotal for promoting the development of a sustainable hydrogen economy.^[^ ^1^ ^]^ Stratiform transition metal dichalcogenides (TMDs) are a typical 2D nanomaterial and have shown substantial progress in terms of catalysis and energy storage applications.^[^ ^2^ ^]^ In the last few years, TMD‐based catalysts have been optimized by introducing additional basal‐plane vacancies and metastable metallic crystal structures to overcome their low concentration of electrocatalytic activity sites and poor intrinsic electroconductibility; thus, the catalysts exhibited much enhanced HER catalytic activity.^[^ ^3^ ^]^ In addition to achieving significant advances in structural designs, other driving forces, such as thermal energy,^[^ ^4^ ^]^ field effect,^[^ ^5^ ^]^ and strain engineering,^[^ ^6^ ^]^ have also been utilized to boost the HER performance of electrocatalysts.^[^ ^7^ ^]^ Because solar energy is an available clean and sustainable energy, solar‐driven photocatalytic and photoelectrochemical water splitting has become one of the hottest topics in materials science.^[^ ^8^ ^]^

Rhenium dichalcogenides (ReX_2_, X = S or Se) are a unique category of compounds that have both distorted triclinic symmetry (1T) crystal structure and excellent photoelectric properties with weak interlayer coupling; these characteristics ensure their excellent hydrogen evolution activities and good response to the whole spectrum of visible light.^[^ ^9^ ^]^ Thus, ReX_2_‐based electrocatalysts for HER are often combined with the use of visible light.^[^ ^10^ ^]^ Although few‐layer ReS_2_ nanosheets (NSs) have been identified as a promising photo‐electro integration platform because they couple catalytic activity with suitable band structure in a single material.^[^ ^11^ ^]^ However, the photoenhanced HER (PE‐HER) performance induced by injection of hot electrons into edge active sites is still limited.^[^ ^12^ ^]^ This is mainly a result of the following: 1) Hot electrons cannot be effectively utilized because of massively quick electron‐hole recombination.^[^ ^13^ ^]^ 2) Low mobility and short diffusion length of hot electrons further increased the difficulty of injecting into edge sites.^[^ ^14^ ^]^ 3) Although most of the optical absorption of ReSe_2_ was in the visible light region, there is still some room for improvement in terms of wide‐spectrum solar response. These factors seriously restrict the synergistic regulations of both catalytic function and optical response properties. To improve both HER and photoresponsive performances, a common strategy is to dope with heterogeneous atoms.^[^ ^15^ ^]^ Recently, Co,^[^ ^16^ ^]^ Cu,^[^ ^17^ ^]^ Zn,^[^ ^18^ ^]^ etc.‐doped TMD nanostructures showed improved HER catalytic activity, but both inevitable change of layered crystal structures and limit doping amount result in a poor use of such strategy. In addition, it was found that replacing the main group atoms (Ge,^[^ ^19^ ^]^ Si,^[^ ^20^ ^]^ Sn,^[^ ^21^ ^]^ etc.) in chalcopyrite can effectively extend the wide‐spectrum solar response, but this does not contribute to electrocatalytic activity.^[^ ^22^ ^]^ Thus, achieving both enhanced HER catalytic activity and improved wide‐spectrum solar response via single element doping is still a challenge.

Herein, to address these issues, the dual‐enhanced performance was achieved in Re_1−_ *_x_*Mo*_x_*Se_2_ through a single method, which can optimize both electrical structure and wide‐spectrum solar absorption, simultaneously. The optimized electrical structure increases the concentration of catalytically active sites and improves electrical conductivity to significantly boost the HER performance. Also, the new intermediate band induced by Mo doping enhances the wide‐spectrum solar absorption in ReSe_2_. Re_0.94_Mo_0.06_Se_2_ NSs achieve low overpotential (η_10_) of 174 mV at −10 mA cm^−2^ with Δ1η_10_ = 65 mV and have a Tafel slope of 112 mV dec^−1^ in acidic medium (0.5 M H_2_SO_4_). Furthermore, η_10_ could be significantly improved to 137 mV under simulated AM 1.5 sun illumination with Δ2η_10_ = 37 mV, and the improvement is five times larger than that for ReSe_2_. This study not only achieves a total of 102 mV (Δ1η_10_ + Δ2*η_10_* = 102 mV) in optimal Re_1−_ *_x_*Mo*_x_*Se_2_, but also paves a general route for achieving a dual‐enhanced PE‐HER process with better HER catalytic activity and broad solar light response via moderate doping.

Re_1−_ *_x_*Mo*_x_*Se_2_ NSs were prepared by a route that combines the conventional solid‐state reaction and ultrasonication‐assisted exfoliation. Powder X‐ray diffraction analysis (**Figure** **1**a) reveals that the highly crystalline 1T′ phase Re_1−_ *_x_*Mo*_x_*Se_2_ (0.03 ≤ *x* ≤ 0.09) was successfully synthesized (JCPDS 52‐0828, *a* = 6.73 Å, *b* = 6.61 Å, *c* = 6.72 Å). Figure 1b shows that the (001) peak in the 2θ area of 13.5°–14.0° is offset in the lower angle direction with an increase in the Mo concentration, and this is because Re ions (≈0.65 Å for Re^4+^) are slightly larger than Mo ions (≈0.63 Å for Mo^4+^).^[^ ^23^ ^]^ To further confirm the crystal structure, Raman characterizations were performed (Figure 1c). In particular, the Raman modes at 117 and 123 cm^‐1^ are ascribed to in‐plane *E*_g_‐like (1–2) vibrational modes, and those located at 157, 171, 179, 190, 194, 207, 217, 230, 238, 246, 259, 280, and 293 cm^‐1^ correspond to out‐of‐plane (*A*_g_‐like, 3–15) vibrational modes. These observations further confirm the formation of 1T′‐ReSe_2_.^[^ ^24^ ^]^ To probe the surface‐chemical states, X‐ray photoelectron spectroscopy (XPS, Figure 1d) was collected. The spin–orbit 4f_7/2_ and 4f_5/2_ orbitals of Re modes were observed at binding energies of 41.31–43.73 eV, respectively, and this matches well with an oxidation state of +4.^[^ ^25^ ^]^ It is worth noting that the Re 4f binding energy decreased about 0.39–0.55 eV with an increase in Mo dopant content because the electronegativity of Mo (2.16) is larger than that of Re (1.90).^[^ ^26^ ^]^ For the Mo 3d spectrum, two split peaks (229.14 and 232.29 eV) are ascribed to the Mo 3d_5/2_ and Mo 3d_3/2_ states (Figure S1a, Supporting Information), respectively.^[^ ^27^ ^]^ Moreover, a doublet of peaks (54.62 and 55.56 eV) is related to the Se 3d_5/2_ and 3d_3/2_ states (Figure S1b, Supporting Information), respectively.^[^ ^28^ ^]^ The proportion of each element (Re/Mo/Se) determined by XPS analysis is close to the designed stoichiometric amounts (Table S1, Supporting Information). Shifts in the XPS peaks and coincident element ratios convincingly demonstrate that Mo was successfully doped into the catalyst.

**Figure 1:**
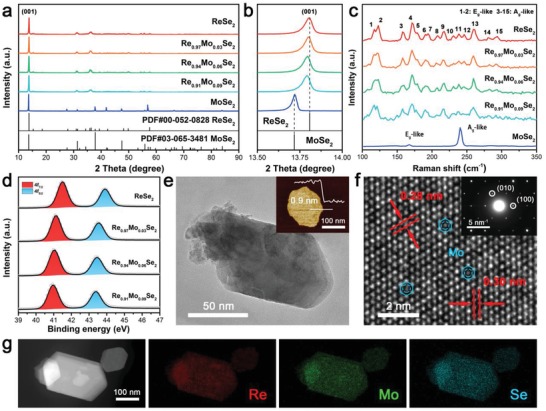
Powder X‐ray diffraction patterns of a) Re_1−_ *_x_*Mo*_x_*Se_2_ and b) the (001) peak in the 2θ area of 13.5°–14.0°. The patterns exhibit a diffraction signal that is offset in the lower angle direction. c) Raman spectra and d) Re 4f XPS spectra of Re_1−_ *_x_*Mo*_x_*Se_2_. e) TEM image and (inset of (e)) atomic force microscopy image, f) high‐resolution TEM image and (inset of (f)) selected area electron diffraction results, and g) energy‐dispersive X‐ray spectroscopy mapping images of as‐exfoliated Re_0.94_Mo_0.06_Se_2_ NSs.

A transmission electron microscopy (TEM) image (Figure 1e) shows that Re_0.94_Mo_0.06_Se_2_ exhibits a typical NS morphology with a length of ≈50–100 nm. The thickness was evaluated using tapping mode atomic force microscopy with an average height of approximately 0.9 nm, as illustrated in Figure 1e, indicating that the NSs consist of about one to two layers (Figures S2 and S3, Supporting Information). As seen in the high‐resolution TEM image shown in Figure 1f, the parallel aligned Re atoms that were in a rhombic arrangement with a distorted 1T structure were partially replaced by Mo atoms that occupied trigonal prismatic sites.^[^ ^29^ ^]^ The crystal lattice fringes are 0.30 and 0.28 nm, which are consistent with the (100) and (010) planes of ReSe_2_, respectively. The selected area electron diffraction pattern clearly demonstrates the singly crystal property of Re_0.94_Mo_0.06_Se_2_ NS, as illustrated in Figure 1f. Moreover, images of energy‐dispersive X‐ray spectroscopy mapping confirm a uniform and homogeneous assignment of the NSs constituents (Figure 1g).

Because Mo doping can optimize the electrical structure, increased catalytically active sites, improved electrical conductivity, and enhanced wide‐spectrum solar response in ReSe_2_ NSs are expected. To verify the enhanced HER performance first, electrocatalytic activities of the various Re_1−_ *_x_*Mo*_x_*Se_2_ NSs were investigated using carbon fiber cloth as a substrate in 0.5 M H_2_SO_4_. All the key electrochemical results after *iR* correction are also listed in **Table** **1**. Among them, Re_0.94_Mo_0.06_Se_2_ NSs display the optimum η of 174 mV to achieve a geometric current density at −10 mA cm^−2^, whereas other Re_1−_ *_x_*Mo*_x_*Se_2_ NSs require η_10_ values of 239 mV (*x* = 0), 212 mV (*x* = 0.03), 187 mV (*x* = 0.09), and 390 mV (*x* = 1) (denoted by the solid line in **Figure** **2**a; Figure S4a, Supporting Information), respectively, and these results demonstrate that appropriate amount of Mo doping could significantly boost the HER performance of ReSe_2_ with a largest reduced overpotential (Δ1η_10_) of 65 mV. From the extrapolation of the linear region of η versus log |*j*| in Figure S4b, Supporting Information, the Tafel slopes of 138, 119, 112, 116, and 219 mV dec^−1^ for ReSe_2_, Re_0.97_Mo_0.03_Se_2_, Re_0.94_Mo_0.06_Se_2_, Re_0.91_Mo_0.09_Se_2_, and MoSe_2_ NSs, respectively, are obtained after *iR* correction. Note that the Tafel slope (112 mV dec^−1^) for Re_0.91_Mo_0.09_Se_2_ suggests that the HER process followed a Volmer–Heyrovsky mechanism.^[^ ^30^ ^]^ Furthermore, the electrochemically active surface area for the HER process was calculated via double‐layer capacitance (*C*_dl_), which was estimated from an analysis of cyclic voltammetry data (Figure S5, Supporting Information). The *C*_dl_ value of Re_0.94_Mo_0.06_Se_2_ NSs (35 mF cm^−2^) is larger than those of ReSe_2_ (15.9 mF cm^−2^), Re_0.97_Mo_0.03_Se_2_ (18.9 mF cm^−2^), Re_0.91_Mo_0.09_Se_2_ (22 mF cm^−2^), and MoSe_2_ (5.32 mF cm^−2^) NSs, demonstrating that Re_0.94_Mo_0.06_Se_2_ NSs have more exposed electrocatalytically activity sites.

**Table 1:** Summary of the electrocatalytic performance parameters for as‐exfoliated Re_1−_ *_x_*Mo*_x_*Se_2_ NSs

Re_1−_ *_x_*Mo*_x_*Se_2_	η_10_ [mV]	Δ2η_10_ [mV]	Tafel slope [mV dec^−1^]	*C*_dl_ [mF cm^−2^]	*R*_ct_ [Ω]
ReSe_2_	239	7	138	15.9	43.5
Re_0.97_Mo_0.03_Se_2_	212	32	119	18.9	39.1
Re_0.94_Mo_0.06_Se_2_	174	37	112	35.0	29.7
Re_0.91_Mo_0.09_Se_2_	187	15	160	22.0	32.7
MoSe_2_	390	0	219	5.32	70.5

**Figure 2:**
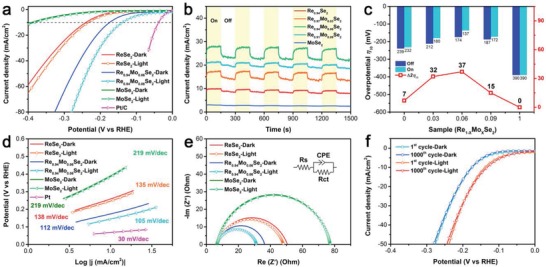
Electrochemical characterization of as‐exfoliated Re_1−_ *_x_*Mo*_x_*Se_2_ NSs. a) Polarization curves, b) *i‐t* curves under intermittent illumination with the on/off cycles of 150 s at the overpotential of 250 mV, c) overpotential (η_10_ and Δ2η_10_), d) corresponding Tafel plots, e) electrochemical impedance spectroscopy Nyquist plots, and f) durability test for 1000 cycles.

Next, to probe another role of Mo doping in improving the photoinduced conductivity, amperometric *i–t* curves for Re_1−_ *_x_*Mo*_x_*Se_2_ (Figure 2b, under AM 1.5 for 150 s of intermittent illumination) at a constant η of 250 mV show variations in the light response as a function of Mo content. Specifically, all of the curves show good reversible behavior in the photocurrent and the largest enhancement is achieved in Re_0.94_Mo_0.06_Se_2_, indicating that there is an optimum band structure that maximizes generation of photogenerated hot electrons. It is, thus, expected that these electrons further promote the HER catalytic activity. To verify the synergistic effects from both an increase in exposed catalytically active sites and additional photogenerated hot electrons caused by Mo doping, Figure 2a and S6, Supporting Information, compare the HER responses (after *iR* correction) of various samples under AM 1.5 illumination. Clearly, the light field further significantly decreased the η value for Re_1−_ *_x_*Mo*_x_*Se_2_. The reduced overpotentials (Δ2η_10_) values for ReSe_2_, Re_0.97_Mo_0.03_Se_2_, Re_0.94_Mo_0.06_Se_2_, Re_0.91_Mo_0.09_Se_2_, and MoSe_2_ NSs under illumination are summarized in Figure 2c. As expected, the optimum photoenhanced HER catalytic activity in Re_0.94_Mo_0.06_Se_2_ (Δ2η_10_ = 37 mV) is superior to other samples, as shown by the volcano‐shaped relationship between HER activity and the level of Mo doping (bottom of Figure 2c). Herein, the largest improvement observed in both Δ1η_10_ (*x* = 0.06) and Δ2η_10_ (*x* = 0.06) convincingly suggests that there is an optimum level of Mo doping to simultaneously utilize the contribution from the optimized electrical structure, which not only results in suitable hydrogen adsorption of free energy, but also increases the number of photogenerated electrons. Note that, Re_0.94_Mo_0.06_Se_2_ was not promoted much in its Tafel slope (ΔTS, ≈7 mV dec^–1^) under illumination (Figure 2d), indicating that the HER mechanism (Volmer–Heyrovsky) did not change after the injection of photogenerated hot electrons.

Electrochemical impedance spectroscopy Nyquist curves (Figure 2e and S4c) matched the Randles circuit, as illustrated as inset in Figure 2e, which showed electron transfer resistance (*R*_ct_) of 43.5, 29.7, and 70.5 Ω for ReSe_2_, Re_0.94_Mo_0.06_Se_2_, and MoSe_2_ NSs, respectively. Because of the faster charge transfer between electrolyte and catalyst in a light field, Re_0.94_Mo_0.06_Se_2_ NSs exhibited a lower *R*_ct_ (24.5 Ω) than in the dark field. The key electrochemical results under illumination are also listed in Table 1. Stability of the catalytic performance over time and cycling is another key indicator to evaluate the catalysts. Chronopotentiometry (*i–t*) curves of Re_0.94_Mo_0.06_Se_2_ under a fixed η (200 mV) reveal a negligible degradation, even after 20 000 s (Figure S4d, Supporting Information). Also, the polarization curves were measured after 1000 cycles (Figure 2f), which were almost overlapped whether in dark or light field, suggesting the excellent long‐term stability.

To deeply investigate the role of Mo doping in Re_0.94_Mo_0.06_Se_2_ NSs, band structure characterizations were performed. UV–vis–NIR diffuse reflectance spectra (**Figure** **3**a) and corresponding band gap spectra (inset in Figure 3a) show that the doped samples exhibit enhanced light‐harvesting capabilities, including extended light absorption (with wavelengths up to ≈1090 nm) and a decreased band gap. The extra absorption peak at ≈1260–1380 nm clearly indicates that there is a new energy level induced by Mo doping. Furthermore, the absorption of Re_0.91_Mo_0.09_Se_2_ NSs at 1000–2000 nm demonstrates that excessive dopant forms additional impurity states.^[^ ^31^ ^]^ Combining the UV photoelectron spectroscopy with the reduction potential of H_2_O, the energy levels of the samples with different doping content are shown in Figure 3b and Figure S7, Supporting Information. It is found that Mo doping not only shifted both the conduction band (CB) and valence band (VB) upward, but also induced the formation of a new intermediate band (IB). This band structure could result in improvement in 1) permitting electron transfer to occur more rapidly into the hydrogen energy level (overpotential), decreasing the reaction barrier; and 2) generating more hot electrons injection.^[^ ^18^ ^]^ Aside from the energy level matching, the electrical conductivity also plays an important role for Re_1−_ *_x_*Mo*_x_*Se_2_ as an integrated electrocatalyst during HER. The carrier density of Re_1−_ *_x_*Mo*_x_*Se_2_ was analyzed by electrochemical Mott–Schottky (M–S) plots in Figure 3c and Figure S8, Supporting Information. The negative slope clearly confirms that Re_1−_ *_x_*Mo*_x_*Se_2_ samples are p‐type semiconductors. According to the proportional relationship with the slope in M–S equation (see Supporting Information), the carrier density gradually increases with an increase in doping concentration, which contributes to a higher in‐plane electrical conductivity.^[^ ^32^ ^]^ Moreover, current–voltage (*I–V*) measurements show that the electrical conductivity of Re_0.94_Mo_0.06_Se_2_ (2.73 × 10^−3^ S cm^−1^) was obviously improved compared to that of ReSe_2_ (2.25 × 10^−3^ S cm^−1^) and MoSe_2_ (1.57 × 10^−5^ S cm^−1^) (Figure S9 and Table S1, Supporting Information). To demonstrate the formation of charged excitons, the emission doublet peak photoluminescence (PL) spectra (Figure S10, Supporting Information) were observed at ≈1.28 and 1.13 eV for ReSe_2_ and Re_0.94_Mo_0.06_Se_2_, respectively, and this suggests that the bound excitons of ReSe_2_ are able to stably combine with unbound electrons to make three‐body bound states (trions) at room temperature.^[^ ^33^ ^]^ To further investigate the effects of doping on the Auger recombination lifetimes of ReSe_2_ and to explore the hot electron dynamics, time‐resolved transient PL spectra (Figure 3d) were recorded.^[^ ^34^ ^]^ Because of poor charge‐transfer capability, ReSe_2_ and MoSe_2_ NSs exhibit a rapid recombination with short PL lifetimes of 2.09 and 2.36 ns, respectively. Longer lifetimes of 3.07, 3.15, and 2.60 were obtained for Re_0.97_Mo_0.03_Se_2_, Re_0.94_Mo_0.06_Se_2_, and Re_0.91_Mo_0.09_Se_2_, respectively, indicating the decreased recombination and effective utilization of hot electrons in ReSe_2_ via moderate Mo doping. Herein, it can be concluded that the optimized band structure resulted in 1) the generation of more hot electrons under wide‐spectrum solar light; 2) extended electron‐hole recombination lifetimes; 3) improved electrical conductivity; and 4) diminished HER reaction process barriers. These contributions lead to significantly photoenhanced HER performance.

**Figure 3:**
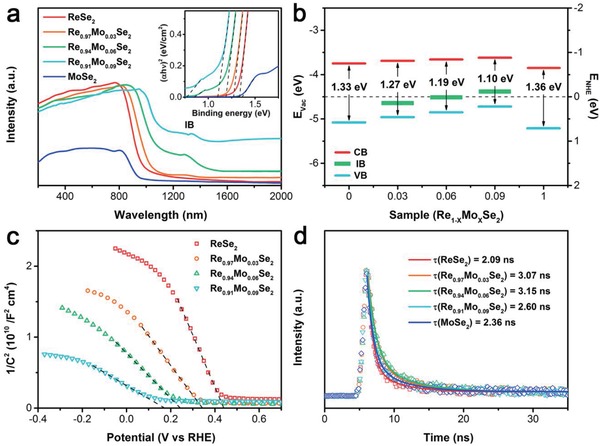
a) UV–vis–NIR absorption spectra, b) energy‐level diagram, c) M–S plots, and d) time‐resolved transient photoluminescence spectra of as‐exfoliated Re_1−_ *_x_*Mo*_x_*Se_2_ NSs.

To further verify the coordination environment of atomic Re, X‐ray absorption near‐edge structure (XANES) and extended X‐ray absorption fine structure (EXAFS) of representative ReSe_2_ and Re_0.94_Mo_0.06_Se_2_ NSs were performed. As shown in Re L_3_‐edge XANES (inset in **Figure** **4**a) and Fourier transformed EXAFS spectra, ReSe_2_ and Re_0.94_Mo_0.06_Se_2_ NSs exhibit high crystallinity. This indicates that Mo atoms were doped into the ReSe_2_ lattice rather than being adsorbed on the surface.^[^ ^35^ ^]^ The positions of the peaks that correspond to Re–Se and Re–Re bonding are located at 2.11 and 2.91 Å, respectively.^[^ ^36^ ^]^ Herein, the number of Se edges in ReSe_2_ can be quantified using the coordination number ratio between Re and Se.^[^ ^37^ ^]^ An increase in Re–Se coordination in Re_0.94_Mo_0.06_Se_2_ indicates that Se edges are effectively exposed when the structure is doped with Mo, which will provide more active sites for the HER process in good agreement with previous electrochemical characterizations results. Moreover, density functional theory calculations were also carried out to investigate the origin of the boost in HER catalytic activity in Re_1−_ *_x_*Mo*_x_*Se_2_ catalyst (see Supporting Information). The hydrogen adsorption free energy (Δ*G*_H*_) is a well‐known indicator for HER catalysts, and the expected Δ*G*_H*_ value is close to 0 eV.^[^ ^38^ ^]^ The hydrogen adsorbed structures of the basal plane and edge models for Re_1−_ *_x_*Mo*_x_*Se_2_ are shown in Figure 4b. The values of Δ*G*_H*_ of the ReSe_2_ and MoSe_2_ planes are 1.72 and 1.98 eV, respectively, indicating the inert surface of ReSe_2_ and MoSe_2_ in agreement with previous reports (Figure 4c).^[^ ^39^ ^]^ After ReSe_2_ is doped with Mo, the calculated Δ*G*_H*_ of basal plane Se site decreased to 0.23 eV, demonstrating that the basal plane of ReSe_2_ is activated by Mo incorporation and the concentration of active sites is further increased. Moreover, the calculated values of Δ*G*_H*_ are 0.43 and 0.59 eV for ReSe_2_ and MoSe_2_ edge sites, respectively. Positive Δ*G*_H*_ values suggest a weak hydrogen sorption process on the Se‐edge during HER. In comparison to ReSe_2_ and MoSe_2_, Re_0.94_Mo_0.06_Se_2_ shows a lower value of Δ*G*_H*_ (0.09 eV), and this proves that regulating the electronic structure is significant for enhancing HER activity. To further understand the band structures of Re_1−_ *_x_*Mo*_x_*Se_2_, the density of states was simulated in Figure 4d. The calculated results indicate that the band gap energy of ReSe_2_ decreased from 1.39 to 0.94 eV with an increase in Mo doping, and this agrees with the extended light response in UV–vis–NIR diffuse reflectance spectra. Because the electrical conductivity plays an important role during electrocatalysis, a reduced band gap for Re_1−_ *_x_*Mo*_x_*Se_2_ will promote HER performance. Moreover, the IB that appears within the band gap of Re_0.94_Mo_0.06_Se_2_ is created by the hybridization of the Re d‐orbital after Mo doping, which is consistent with the extra absorption photons.^[^ ^40^ ^]^ Figure 4e–g shows excitation and recombination paths of hot electrons in ReSe_2_ with different concentrations of Mo doping. ReSe_2_ has strong Coulomb interactions, and it usually exhibits multiexciton states with long Auger recombination lifetimes.^[^ ^13^ ^]^ In ReSe_2_ NSs, hot electrons can be excited into the CB and nonradiatively transfer energy to an extra charge (Figure 4e).^[^ ^41^ ^]^ With low doping concentrations (Figure 4f), doped Mo atoms introduce impurity states near the VB edge, and this leads to the absorption of additional lower energy photons via a two‐step process.^[^ ^42^ ^]^ However, excessive Mo doping leads to a wider IB (Figure 4g), and this generates recombination centers in the band gap. Therefore, electron‐hole pairs are induced to recombine faster via indirect recombination, and this will reduce the lifetimes of the hot electrons.^[^ ^43^ ^]^

**Figure 4:**
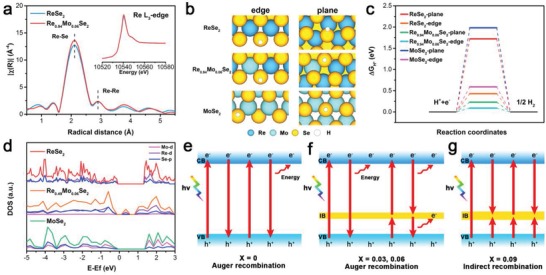
XANES spectra (inset in panel (a)) and a) EXAFS spectra of the as‐exfoliated ReSe_2_ and Re_0.94_Mo_0.06_Se_2_ NSs. b) Density functional theory schematic diagram. c) HER free‐energy diagram. d) Density of states plots for monolayers of ReSe_2_, Re_0.94_Mo_0.06_Se_2_, and MoSe_2_. e–g) Schematic diagrams of the separation and recombination process for an electron‐hole in Re_1−_ *_x_*Mo*_x_*Se_2_.

In summary, for the first time, exposed catalytically active sites, improved electrical conductivity, and enhanced wide‐spectrum solar response were simultaneously achieved in ReSe_2_ NSs via a moderate Mo doping to enhance the catalytic activity toward PE‐HER. By tuning the content of Mo doping, there exist an optimized electrical structure to enhance the HER performance first, and when irradiated under solar light, the generated hot electrons are efficiently injected into catalytic sites to further improve the HER catalytic activity, resulting a dual‐enhanced PE‐HER performance. The synergistic modulations of these two factors result in a superior PE‐HER electrocatalytic activity in optimal Re_0.94_Mo_0.06_Se_2_ NSs. The overpotential (η_10_) is first reduced from 239 to 174 mV with Δ1η_10_ of 65 mV, and the Tafel slope is 112 mV dec^−1^. Then, a further reduced value of 37 mV (Δ2η_10_) was achieved under simulated AM 1.5 sun illumination. Thus, the total improvement (Δη_10_ = Δ1η_10_ + Δ2η_10_) of about 102 mV was obtained toward PE‐HER in Re_0.94_Mo_0.06_Se_2_. This work may present an avenue for further research regarding highly efficient TMD electrocatalysts for PE‐HER.

## Conflict of Interest

The authors declare no conflict of interest.

## Supporting information

Supporting Information
