# Supplementary material for: Dual‐Enhanced Doping in ReSe2 for Efficiently Photoenhanced Hydrogen Evolution Reaction
Source: Adv Sci (Weinh). 2020 Mar 16;7(9):2000216. doi: 10.1002/advs.202000216 (PMC7201260; doi:10.1002/advs.202000216)
Supplement: Supplementary file 1 — Supporting Information [file ADVS-7-2000216-s001.pdf]

Copyright WILEY-VCH Verlag GmbH & Co. KGaA, 69469 Weinheim, Germany, 2020.

ADVANCED SCIENCE
Open Access

Supporting Information

for *Adv. Sci.,* DOI: 10.1002/advs.202000216

Dual-Enhanced Doping in $ReSe_2$ for Efficiently Photoenhanced Hydrogen Evolution Reaction

Ran Wang, Jiecai Han, Ping Xu, Tangling Gao, Jun Zhong, Xianjie Wang, Xinghong Zhang, Zhijun Li, Lingling Xu, and Bo Song**

Copyright WILEY-VCH Verlag GmbH & Co. KGaA, 69469 Weinheim, Germany, 2020.

Supporting Information

Dual-Enhanced Doping in $ReSe_2$ for Efficiently Photo-Enhanced Hydrogen Evolution Reaction

Ran Wang, Jiecai Han, Ping Xu, Tangling Gao, Jun Zhong, Xianjie Wang, Xinghong Zhang, Zhijun Li, Lingling Xu, and Bo Song**

Experimental Section

Materials: The selenium powder (99.99 %), rhenium powder (99.999 %), and molybdenum powder (99.999 %) were purchased from Alfa Aesar. Benchmark 20 wt % Pt/C catalysts were purchased from Aladdin. All chemicals were of analytical grade and were used as received without purification, unless otherwise specified.

Synthesis of $Re_{1-x}Mo_xSe_2$ NSs: Bulk $Re_{1-x}Mo_xSe_2$ (x = 0, 0.03, 0.06, 0.09, 1) samples were prepared by traditionally solid-state reactions with stoichiometric amounts of Re, Mo, and Se powders (total mass of 1 g). Before the solid-state reactions, the elemental powder was grinded uniformly to make the reaction more sufficient. The elemental powders were sealed into quartz tubes under a dynamic vacuum of 5.5×10^{-4} Pa. The seals samples were heated in a muffle furnace from room temperature to 900 °C with a heating rate of 1 °C min^{-1}. After it was maintained at 900 °C for 120 h, the furnace was cooled to room temperature naturally, and a series of bulk samples were obtained. $Re_{1-x}Mo_xSe_2$ NSs were obtained from an ultrasonication-assisted exfoliation technique. Bulk $Re_{1-x}Mo_xSe_2$ (50 mg) was added to the water-ethanol solution (100 mL) and ground for 120 min to ensure that large particles were sufficiently crushed into small ones. The dispersion liquid was sonicated for 1 h (500 W) at room temperature in an ultrasonic washer. The mixture was centrifuged and the supernatant was decanted. Then, the supernatant was put in a typical probe sonication lasted for 12 h, and the sonication power was adjusted to 900 W with an on/off cycle of 5/3 s to get the exfoliated

$Re_{1-x}Mo_xSe_2$ NSs. The uniform solution was centrifuged in 3000 r/min first. And then, the decanted supernatant was centrifuged again in 9000 r/min and the $Re_{1-x}Mo_xSe_2$ NSs were obtained. The samples were washed with absolute ethanol and water sequentially. Finally, the $Re_{1-x}Mo_xSe_2$ NSs were dried at 60 °C for 12 h in a vacuum oven.

Structural Characterization: Powder XRD measurements were performed on a Rigaku D/max 2500 X-ray diffractometer using the Cu Kα radiation (λ = 1.5406 Å). Raman spectra were collected on a Renishaw inVia confocal micro-Raman spectroscopy system using a TE air-cooled 576 × 400 CCD array with a 532 nm excitation laser. X-ray photoelectron spectra (XPS) were recorded on an ESCALAB MKII spectrometer using an Al Kα excitation source. A JEOL ARM 200F transmission electron microscope and a field-emission gun (FEG) scanning electron microscope (JEOL 6500 SEM) were used to characterize the morphology and structure of the nanosheet materials. All TEM images were processed by using the DigitalMicrograh. Because Mo ions (~0.63 Å for Mo^{4+}) were slightly smaller than Re ions (~0.65 Å for Re^{4+}) and occupied trigonal prismatic sites, we distinguished Mo atoms by atomic size and atomic spacing identification.[1] The thickness of the $Re_{1-x}Mo_xSe_2$ NSs was analyzed by atomic force microscopy (AFM) on a Bruker DI MultiMode-8 system. UV-Vis-NIR absorption spectra was recorded on a PerkinElmer Lambda 950 UV/Vis-NIR spectrophotometer. The energy band was evaluated by the ultraviolet photoemission spectroscopy (UPS) in the vacuum of less than 10^{-7} Pa and the energy step size is 0.02 eV. The $Re_{1-x}Mo_xSe_2$ samples were pressed into disks with a diameter (D) of 10 mm and a thickness (L) of ~0.5 mm. *I–V* characteristics were measured in air at room temperature using a KEITHLEY 2602B System SourceMeter. The conductivity according to the following equation:

$$k = (IL)/(0.25\pi D^2 V) \quad (1)$$

The room photoluminescence (PL) spectra (excited by 325 nm illumination) were record on a RenishawRM3000 Micro-Raman system. The time-resolved transient photoluminescence

(TRPL) spectra were recorded on an Edinburgh instruments as LifeSpec II. The extended X-ray absorption fine structure (EXAFS) measurements of Re L_3-edge were performed at the XAFCA beamline of Shanghai Synchrotron Radiation Facility (SSRF). Data processing and fitting were carried out using the Demeter software package.

Electrochemical Measurements: Electrochemical measurements were performed with a standard three-electrode setup (CH Instruments) using Ag/AgCl (in 3.5 M KCl solution) as the reference electrode, a graphite rod (Alfa Aesar, 99.9995%) as the counter electrode, and the clean carbon fiber cloth coated with drop-cast $Re_{1-x}Mo_xSe_2$ catalysts as the working electrode. The catalyst was ultrasonically dispersed in a water-ethanol solution (v/v=3: 7) containing 0.1 wt % Nafion, and a drop of the catalyst (10 μL, 5 μg μL^{-1}) was then transferred onto the carbon fiber cloth electrode with a geometric area of 1 cm^2. The amount of deposited catalyst was calculated to be ~50 μg. All measurements were performed in H_2-saturated H_2SO_4 (0.5 M) aqueous solution. The working electrode was irradiated at the front side with simulated sunlight emitted from a Xe lamp with an AM 1.5G filter. Cyclic voltammograms taken at various scan rates (20–180 mV s^{-1}) were collected in the 0.1–0.2 V vs. RHE range and used to estimate the double-layer capacitance. The EIS measurements were carried out at 200 mV overpotential in the frequency range from 10^6 to 0.1 Hz. To better compare the catalytic activity of the different catalysts, we used the series resistance determined from EIS experiments to correct the polarization measurements and subsequent Tafel analysis for the *iR* loss. The electrochemical stability of the catalyst was evaluated by cycling the electrodes for 1,000 times. All potentials were referenced to a reversible hydrogen electrode (RHE). Mott-Schottky (M-S) plots were obtained at an AC frequency of 1.0 kHz. The plots provide information about the carrier density according to the following equation:

$$1/C^2 = (2/e\varepsilon\varepsilon_0 N_D A^2)[(V - V_{FB}) + k_B T/e] \quad (2)$$

C is the specific capacity, ε is the dielectric constant of the photoanode, ε_0 is the vacuum electric permittivity, N_D is the carrier density, A is the area, V is the applied potential, V_{FB} is

the flat band potential, k_B is the Boltzmann constant, T is the absolute temperature, and e is the elementary charge.

Computational Methods: All the density functional theory (DFT) calculations were performed by using the Vienna Ab-initio Simulation Package (VASP), employing the Projected Augmented Wave (PAW) method.[2] The revised Perdew-Burke-Ernzerhof (RPBE) functional was used to describe the exchange and correlation effects.[3] In all the cases, the cutoff energy was set to be 450 eV.[4] The 2H-$MoSe_2$, 1T'-$ReSe_2$, and 1T'- $Re_{0.94}Mo_{0.06}Se_2$ were simulated as single-layered structure.[5] A 4×4 supercell was employed in all the calculations.[6] The Monkhorst-Pack grids were set to be 4×4×1 and 9×9×1 for computing the free energy and density of states (DOS) calculations, respectively.[7] At least 18 Å vacuum layer was applied in z-direction of the slab models, preventing the slabs from vertical interactions.

The descriptor proposed by Norskov et al. was used to describe the HER activity on a given catalyst surface, where the free energy of hydrogen adsorption (ΔG_{H*}) was considered as the key parameter determining the HER activity.[8] For HER in acidic solutions, it is a two-step process and involves only one reaction intermediate, the chemisorbed H atom. The free energy of the adsorbed hydrogen is defined as:

$$\Delta G_{H*} = \Delta E_H + \Delta E_{ZPE} - T\Delta S_H \quad (3)$$

where ΔE_H is the hydrogen binding energy, ΔE_{ZPE} is the zero point energy difference between adsorbed hydrogen and gaseous hydrogen, and $T\Delta S_H$ is the corresponding entropy difference between these two states.[9] According to previous studies,[10] here we used a 0.24 eV value to represent the correction of zero point energy and entropy of hydrogen state.

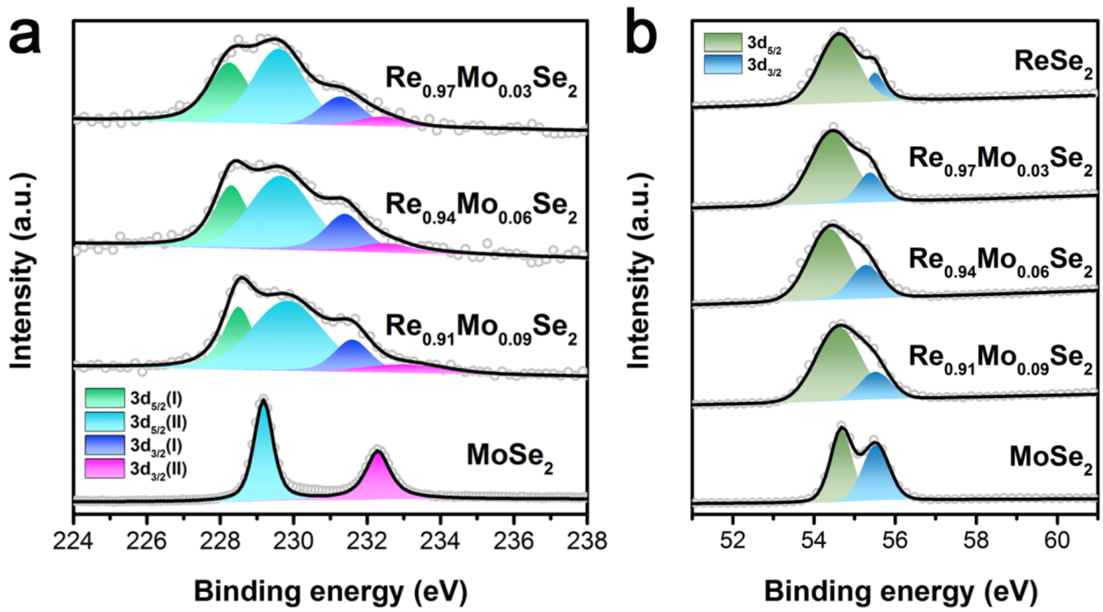

Figure S1. (a) Mo 3d and (b) Se 3d XPS spectra of as-exfoliated $Re_{1-x}Mo_xSe_2$ NSs.

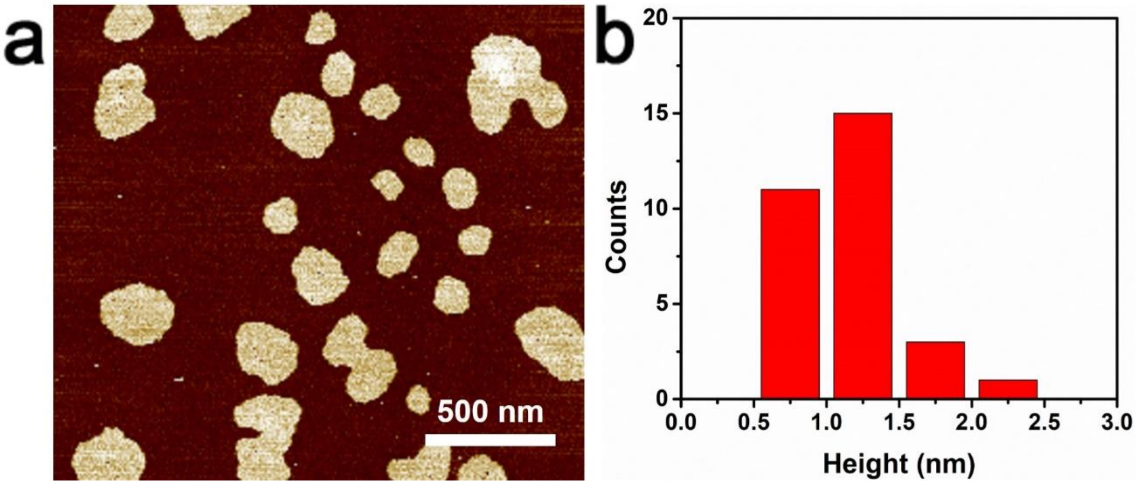

Figure S2. (a) AFM image of as-exfoliated $Re_{0.94}Mo_{0.06}Se_2$ NSs, (b) statistical analysis of the height measured from AFM images.

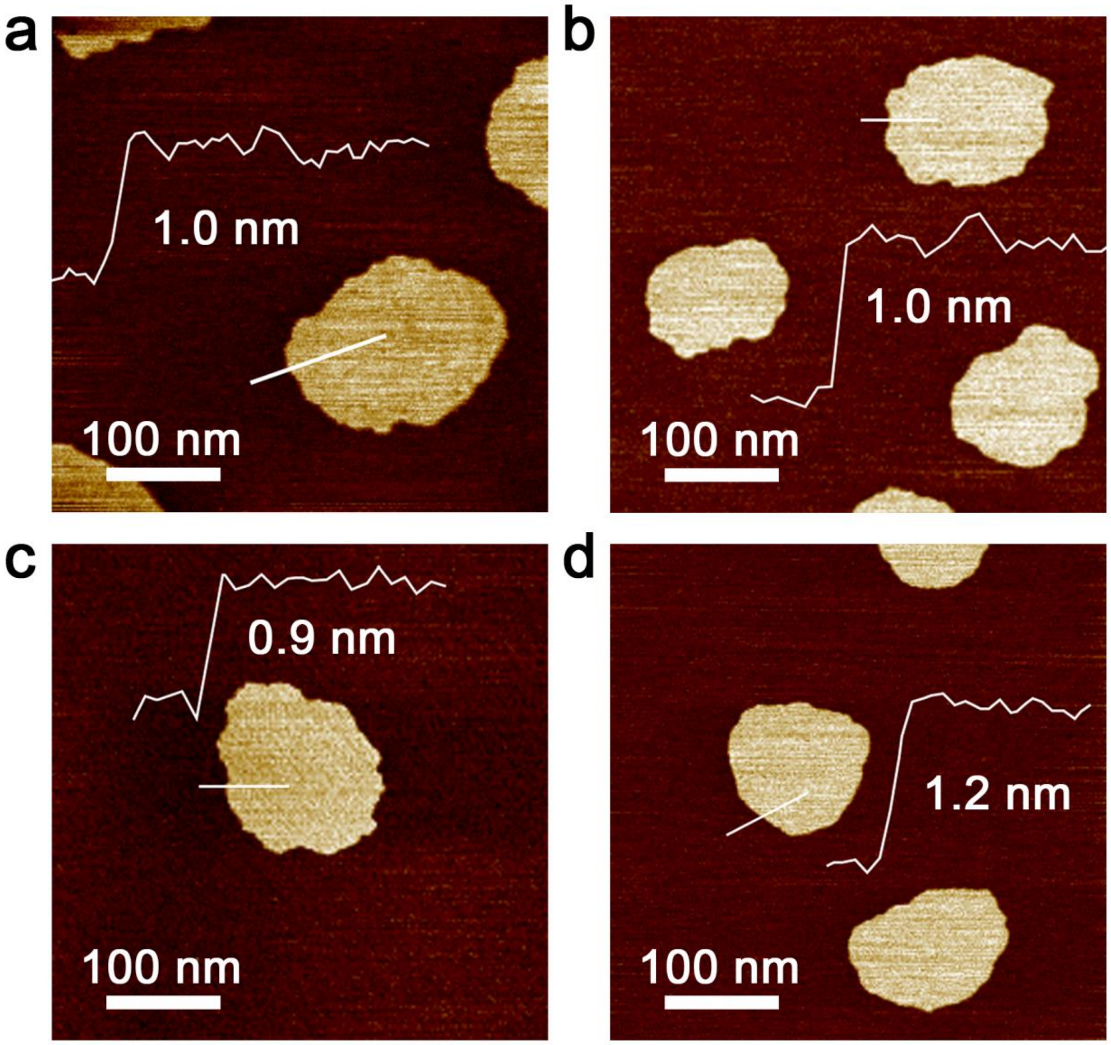

Figure S3. AFM images of as-exfoliated (a) $ReSe_2$, (b) $Re_{0.97}Mo_{0.03}Se_2$, (c) $Re_{0.91}Mo_{0.09}Se_2$, and (d) $MoSe_2$ NSs.

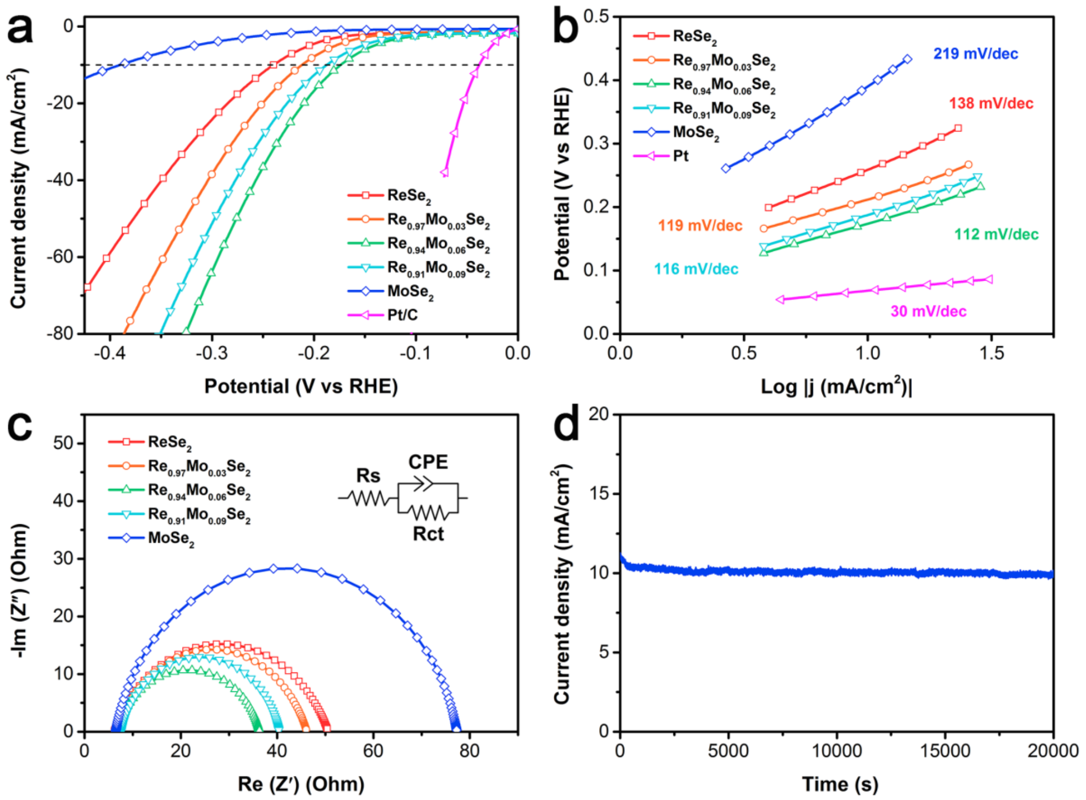

Figure S4. Electrochemical characterization of as-exfoliated $Re_{1-x}Mo_xSe_2$ NSs. (a) Polarization curves, (b) corresponding Tafel plots, (c) EIS Nyquist plots, and (d) amperometric *i-t* curves of $Re_{0.94}Mo_{0.06}Se_2$ NSs.

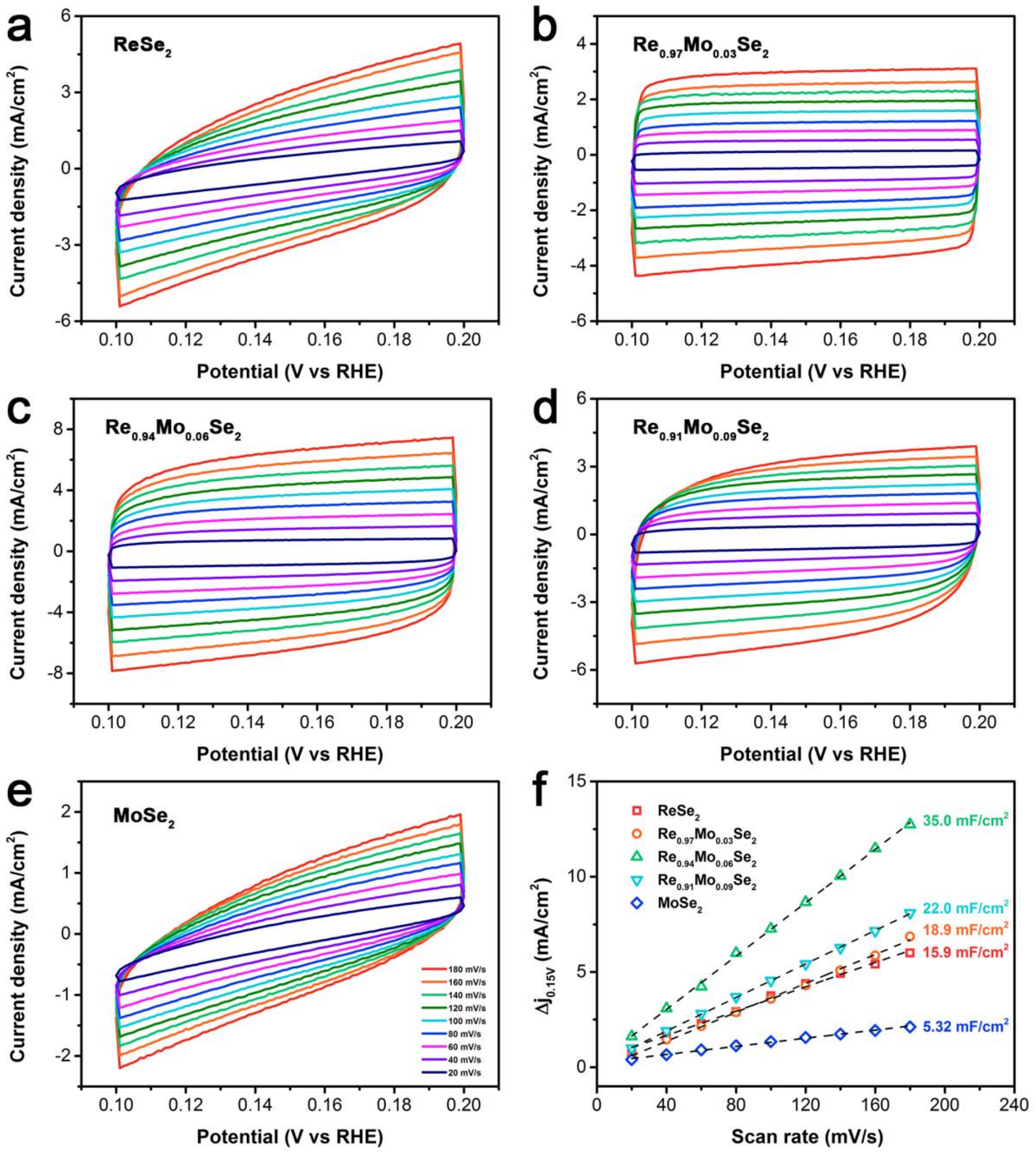

Figure S5. Cyclic voltammetry curves of (a) $ReSe_2$, (b) $Re_{0.97}Mo_{0.03}Se_2$, (c) $Re_{0.94}Mo_{0.06}Se_2$, (d) $Re_{0.91}Mo_{0.09}Se_2$, and (e) $MoSe_2$ NSs under different scan rates in the region of 0.1-0.2 V vs. RHE, (f) double-layer capacitance (C_{dl}) of as-exfoliated $Re_{1-x}Mo_xSe_2$ NSs.

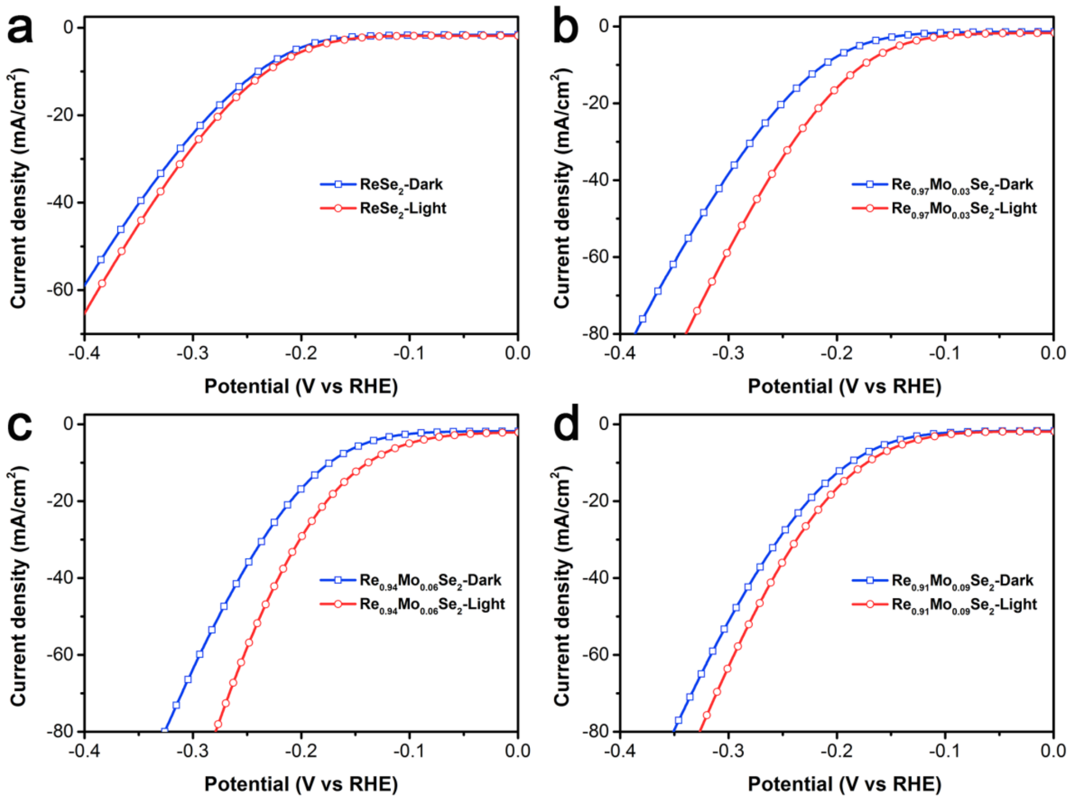

Figure S6. Polarization curves of (a) $ReSe_2$, (b) $Re_{0.97}Mo_{0.03}Se_2$, (c) $Re_{0.94}Mo_{0.06}Se_2$, and (d) $Re_{0.91}Mo_{0.09}Se_2$ NSs in dark and light fields.

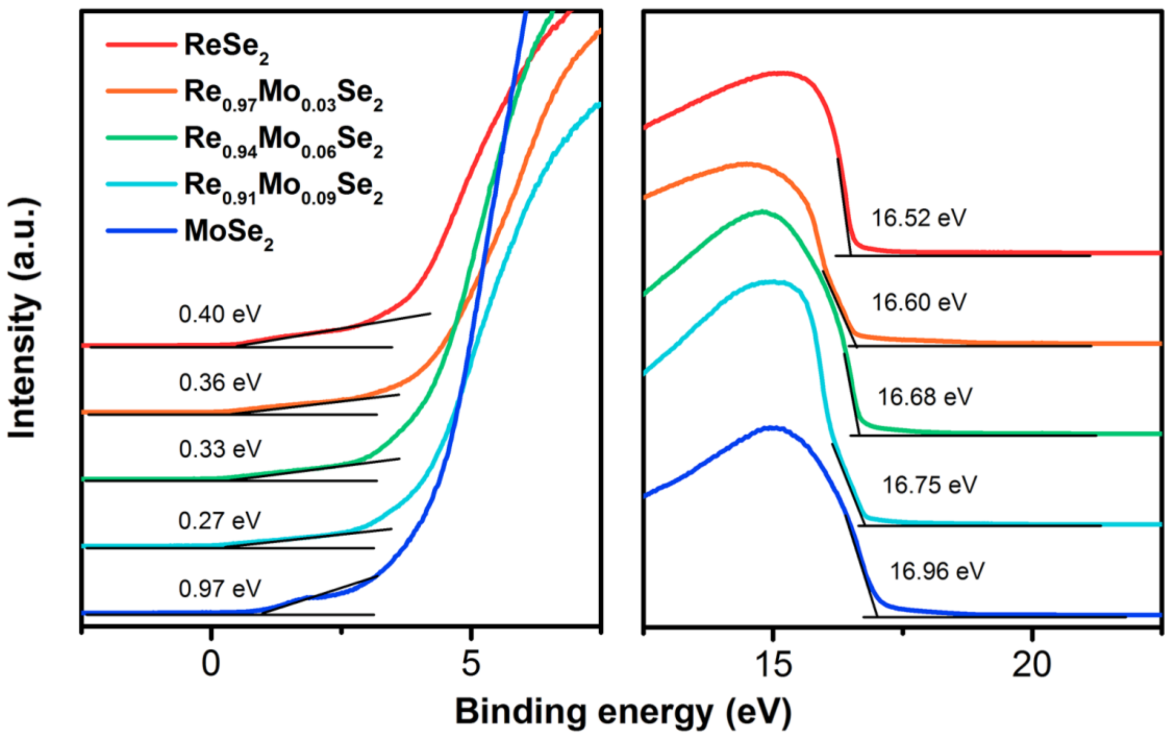

Figure S7. UPS of as-exfoliated $Re_{1-x}Mo_xSe_2$ NSs.

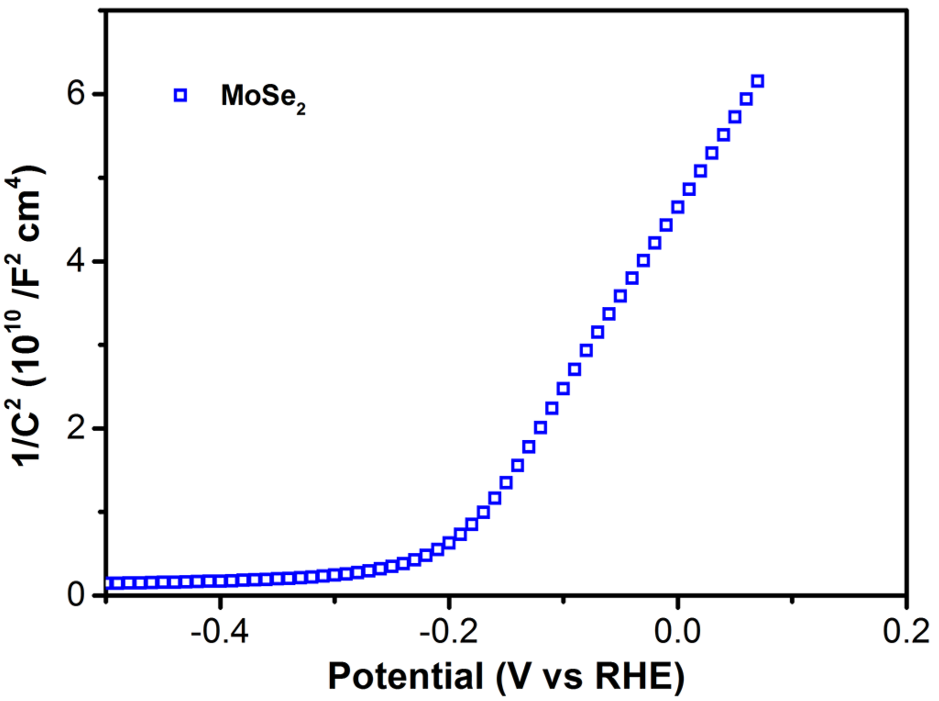

Figure S8 M-S plots of as-exfoliated $MoSe_2$ NSs.

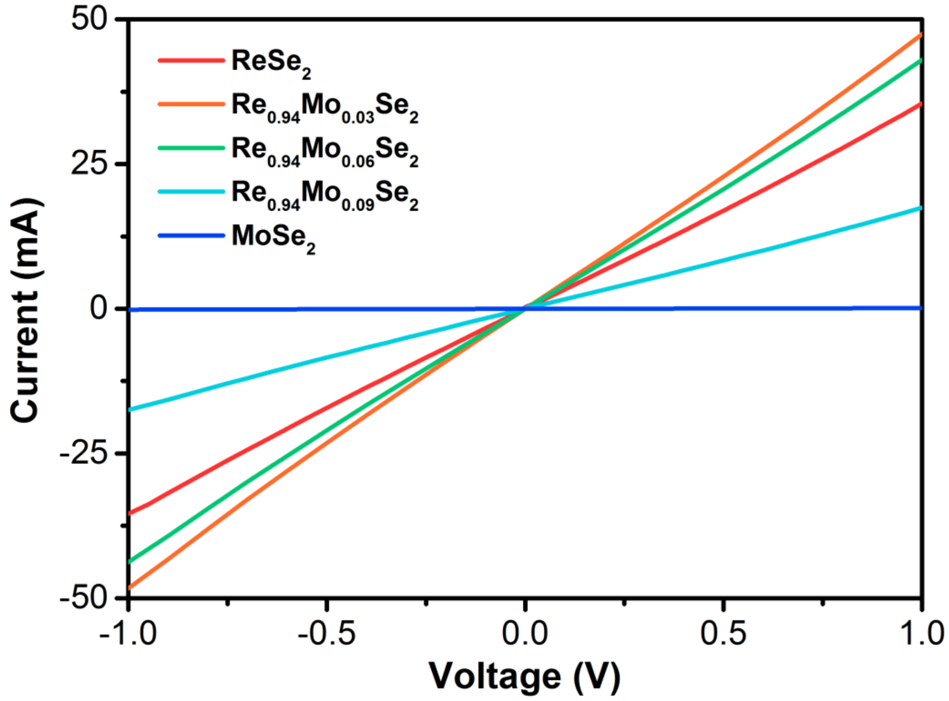

Figure S9 ***I-V*** **measurements of as-exfoliated $Re_{1-x}Mo_xSe_2$ samples.**

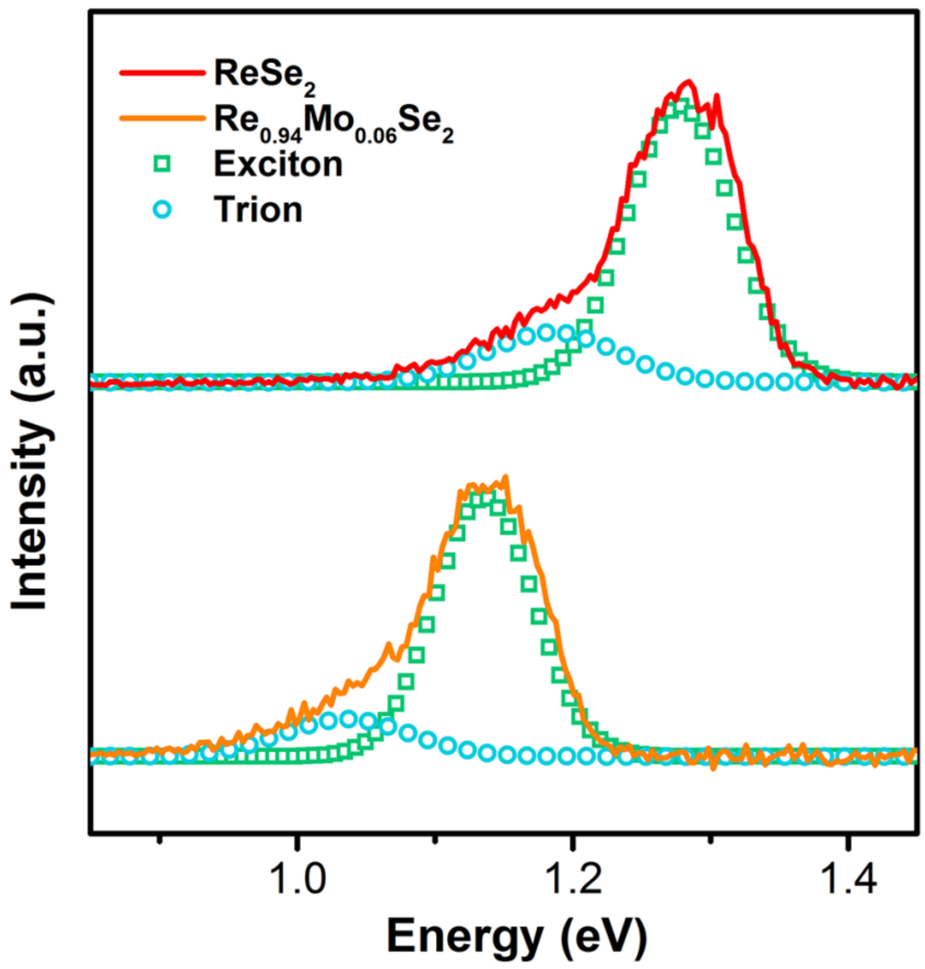

Figure S10 PL spectrum of as-exfoliated $ReSe_2$ and $Re_{0.94}Mo_{0.06}Se_2$ NSs.

Table S1. Summary of the conductivity and atomic ratio for as-exfoliated $Re_{1-x}Mo_xSe_2$ NSs.

$Re_{1-x}Mo_xSe_2$	σ [S cm^{-1}]	Atomic ratio of Re/Mo/Se by XPS
$ReSe_2$	2.25×10^{-3}	1.08:2
$Re_{0.97}Mo_{0.03}Se_2$	3.01×10^{-3}	0.98:0.02:2
$Re_{0.94}Mo_{0.06}Se_2$	2.73×10^{-3}	0.94:0.06:2
$Re_{0.91}Mo_{0.09}Se_2$	1.11×10^{-3}	0.92:0.09:2
$MoSe_2$	1.57×10^{-5}	1.13:2

References

[1] N. Al-Dulaimi, D. J. Lewis, X. L. Zhong, M. Azad Malik, P. O’Brien, *J. Mater. Chem. C* **2016**, 4, 2312

[2] a) B. Hammer, L. B. Hansen, J. K. Nørskov, *Phys. Rev. B.* **1999**, 59, 7413; b) Y. Zheng, Y. Jiao, A. Vasileff, S.-Z. Qiao, *Angew. Chem. Int. Ed.* **2018**, 57, 7568; c) W. Wu, C. Niu, C. Wei, Y. Jia, C. Li, Q. Xu, *Angew. Chem. Int. Ed.* **2019**, 58, 2029.

[3] a) G. Kresse, J. Furthmüller, *Comput. Mater. Sci* **1996**, 6, 15; b) Y. Jiao, F. Ma, J. Bell, A. Bilic, A. Du, *Angewandte Chemie* **2016**, 128, 10448.

[4] S.-Z. Yang, Y. Gong, P. Manchanda, Y.-Y. Zhang, G. Ye, S. Chen, L. Song, S. T. Pantelides, P. M. Ajayan, M. F. Chisholm, W. Zhou, *Adv. Mater.* **2018**, 30, 1803477.

[5] Y. Zhou, E. Song, J. Zhou, J. Lin, R. Ma, Y. Wang, W. Qiu, R. Shen, K. Suenaga, Q. Liu, J. Wang, Z. Liu, J. Liu, *ACS Nano* **2018**, 12, 4486.

[6] A. Luque, A. Martí, *Phys. Rev. Lett.* **1997**, 78, 5014.

[7] D. Hu, T. Zhao, X. Ping, H. Zheng, L. Xing, X. Liu, J. Zheng, L. Sun, L. Gu, C. Tao, D. Wang, L. Jiao, *Angew. Chem. Int. Ed.* **2019**, 58, 6977.

[8] X. Ren, W. Wang, R. Ge, S. Hao, F. Qu, G. Du, A. M. Asiri, Q. Wei, L. Chen, X. Sun, *Chem. Commun.* **2017**, 53, 9000.

[9] Y. Ouyang, C. Ling, Q. Chen, Z. Wang, L. Shi, J. Wang, *Chem. Mater.* **2016**, 28, 4390.

[10] J. K. Nørskov, T. Bligaard, A. Logadottir, J. R. Kitchin, J. G. Chen, S. Pandelov, U. Stimming, *J. Electrochem. Soc.* **2005**, 152, J23.
